# Association between attention deficit hyperactivity disorder and aggression subscales in adolescents

**DOI:** 10.1002/brb3.2030

**Published:** 2021-01-13

**Authors:** Hee Jeong Yoo, Ji Min Han, Kitai Kim, Gonjin Song, Jeong Yee, Jee Eun Chung, Kyung Eun Lee, Hye Sun Gwak

**Affiliations:** ^1^ College of Pharmacy and Graduate School of Pharmaceutical Sciences Ewha Womans University Seoul Republic of Korea; ^2^ Department of Pharmacy National Medical Center Seoul Republic of Korea; ^3^ College of Pharmacy Chungbuk National University Cheongju Republic of Korea; ^4^ Department of Communication Honam University Gwangju Republic of Korea; ^5^ College of Pharmacy, Institute of Pharmaceutical Science and Technology Hanyang University Ansan Republic of Korea

**Keywords:** adolescent, aggression, anger, Attention Deficit Hyperactivity Disorders, hostility

## Abstract

**Introduction:**

The aim of this study is to identify the association between Attention Deficit Hyperactivity Disorder (ADHD) proneness and aggressive propensity in adolescents.

**Methods:**

A quantitative, large‐scale, cross‐sectional study was performed from April to May 2016 in Korea. The survey questionnaire included overall health behaviors, as well as scales for assessing ADHD proneness (revised short form of the Conners‐Wells Adolescent Self‐Report Scale; CASS[S]) and aggressive behavior (Buss‐Perry Aggression Questionnaire; BPAQ) in adolescents. Area under the receiver operator characteristic (AUROC) curves was constructed to determine the cut‐off value of total aggression score for discriminating ADHD proneness.

**Results:**

A total of 2,432 students participated in the survey, and 1,872 of them completed the questionnaire, indicating a response rate of 77.0%. Based on CASS(S), 33 (1.8%) subjects were classified as the ADHD group. AUROC curve analysis showed that a score of 68.5 points had higher sensitivity (83.3%) and specificity (69.4%) to discriminate ADHD proneness. ADHD proneness was significantly associated with higher aggression subdomain scores (physical, verbal, anger, and hostility). Especially, anger and hostility had a stronger relationship with ADHD proneness than did physical and verbal aggression. A multivariable analysis demonstrated that ADHD proneness was significantly related to body mass index in the top 10% of the study population, alcohol consumption, gastrointestinal trouble, daytime sleepiness, and total aggression score of 68.5 points or higher. Adolescents who had total aggression scores of 68.5 points or higher showed a 9.8‐fold (95% confidence interval [CI] 3.3–28.8) higher risk of ADHD compared with those who had scores less than 68.5 points.

**Conclusions:**

Our results demonstrated that ADHD proneness was significantly associated with aggression propensity. In particular, anger and hostility were more closely associated with ADHD proneness than were other aggression subdomains.

## Graphical abstract

This large‐scale cross‐sectional study showed that ADHD proneness was associated with aggression propensity in adolescents. In subdomains of aggression, anger and hostility, which are inconspicuous behaviors, showed stronger association with ADHD tendency than did other aggression subdomains.

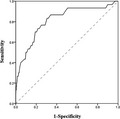

## 1 INTRODUCTION

Attention Deficit Hyperactivity Disorder (ADHD) is a major obstacle to learning and life guidance at school, with relatively high prevalence in childhood and adolescence. ADHD refers to persistent and recurrent disturbances in age‐appropriate behaviors within the two domains of attention deficit and hyperactivity/impulsivity, which cause maladjustment in emotional and social aspects, as well as cognitive and behavioral aspects (Yun & Kim, 1999).

The prevalence rate of ADHD is still increasing. The National Health Interview Survey in the United States showed a 67% rise in ADHD prevalence between 1997 (6.0%) and 2016 (12.1%) among children and adolescents ages 4 to 17 (Xu et al., 2018). When comparing the prevalence of adolescents and children, the prevalence among adolescents ages 12 to 17 was higher than that of children ages 4 to 11, and the ADHD rate of children decreased while that of adolescents increased.

Although the Diagnostic and Statistical Manual of Mental Disorders (DSM–5) typically presents hyperactivity, impulsivity, and inattention as ADHD‐related symptoms, the manifestations of ADHD are highly heterogeneous (Luo et al., 2019). Among the symptoms of ADHD, hyperactivity–impulsivity is known to be related to aggressive behavior, rule‐breaking, and extraversion, while inattention is known to be related to depression, slower cognitive task performance, and introversion (Martel et al., 2011). A previous study reported that impulsivity related with ADHD also caused extreme expression of anger (Gitta et al., 2015).

School violence is an issue in adolescence. A previous study demonstrated that ADHD was a risk factor for school violence (Timmermanis & Wiener, 2011). Hyperactivity and impulsive ADHD can be a major cause of bullying, especially for boys, because of aggressive and rebellious behaviors (Erhardt & Hinshaw, 1994; Lee & Hwang, 2013). Afflicted adolescents are often more hostile and aggressive than their peers, and they have problems with antisociality, depression, and anxiety (Flannery et al., 2004). In addition, they experience psychological problems, such as humiliation, hostility, revenge, anxiety, and fear, as well as emotional issues, such as depression and low self‐esteem (Bonanno & Hymel, 2013). On the other hand, children that exhibit attention deficit via ADHD face difficulties in socializing with their peers, resulting in higher risks of becoming school violence victims (Liu et al., 2017; Ghossoub et al., 2017). Children with ADHD tend to be four times more likely to act as offenders and ten times more likely to become victims than typical children (Holmberg & Hjern, 2008). Because adolescence is a critical period for undergoing social, emotional, and physical changes and forming self‐perception, school violence in adolescence can negatively affect both the perpetrator and the victim (Crocker et al., 2006).

Oppositional defiant disorder (ODD) and conductive disorder (CD), which are classified as disruptive behavior disorders, coexist in ADHD patients at a high rate of 45%–84%, and 15%–56%, respectively (Barkley, 2014; Biederman et al., 1991; Larson et al., 2011). A study revealed that individuals with ADHD and CD in childhood showed increased physical aggression and anger in adolescence, whereas those with ADHD and ODD in childhood exhibited elevated levels of verbal aggression and anger in adolescence (Harty et al., 2009).

Previously, aggression has been classified in a variety of ways. For example, the Buss‐Perry Aggression Questionnaire, one of the most widely used instruments to access aggression, classified aggression into four types; physical aggression, verbal aggression, anger, and hostility (Buss & Perry, 1992; Reyna et al., 2011). In the questionnaire, each individual component represented the instrumental components (physical and verbal aggression), emotional component (anger), and cognitive component (hostility) of aggression.

The purpose of aggression is known to be the key factor in the dichotomy between predative versus. defensive aggression (McKay & Halperin, 2001), while divisions between proactive versus. reactive (Dodge & Coie, 1987) and instrumental versus. hostile (Atkins & Stoff, 1993) aggression depend on the intent and emotional states of aggression. In addition, while premeditated aggression has been defined as aggressive behavior with a certain pre‐conceived goal that is achievable through implementing specific means, impulsive aggression is associated with an uncontrollable emotional state at the time of aggression (Dodge, 1991). Impulsivity and hyperactivity in ADHD children are often evident during early childhood; unlike hyperactivity, impulsivity tends to persist and pervades diverse functions in throughout different stages of development (McKay & Halperin, 2001).

It has been reported that aggression was a major source of impaired levels of inattention and hyperactivity and frequently represented the trigger for initial referral of ADHD assessment (Jensen et al., 2007; King & Waschbusch, 2010). Also, studies showed that aggressive behavior associated with ADHD eventually resulted in important consequences regarding the functioning as an individual and could contribute to adverse outcomes associated with ADHD due to the increase in risk of peer rejection (Evans et al., 2015; Jester et al., 2005, 2008).

Despite their established association, the relationship between ADHD symptoms (cognition, hyperactivity, and conduct) and aggression subscales (physical aggression, verbal aggression, anger, and hostility) has been assessed in only a handful of previous studies. Therefore, the initial aim of this study was to examine the association between ADHD proneness and aggression propensity, using subscales. The secondary aim was to identify the cut‐off value of aggression scores to discriminate ADHD proneness, thereby allowing for targeted intervention of ADHD proneness.

## 2 MATERIALS AND METHODS

### 2.1 Participants

From April to May 2016, this cross‐sectional study was performed with students ages 11 to 19 from middle schools (7th‐9th grades) and high schools (10th‐12th grades) in Gwangju, Korea. With the assistance of statisticians from the Office of Education in the region, six clusters from Gwangju were formed based on sociodemographic characteristics. The questionnaire was distributed during researchers’ on‐site visit to schools. Each participant voluntarily completed the survey. After survey completion, a unique study identification was assigned to each participant in order to ensure confidentiality and anonymity. This study was approved by the Ethics Committee and Institutional Review Board of the Honam University, Korea (IRB number: 1041223–201510‐HR‐090–01). Informed consents were obtained from students’ parents. All procedures performed in this study involving human participants were in accordance with the ethical standards of the ethics committees, which approved the study, and with the 1964 Helsinki declaration and its later amendments or comparable ethical standards.

### 2.2 Measurements

The survey was comprised of the revised short form of the Conners‐Wells Adolescent Self‐Report Scale (CASS[S]) and Buss‐Perry Aggression Questionnaire (BPAQ) (Buss & Perry, 1992; Kollins & Epstein, 2014). A standard instrument for ADHD assessment, CASS(S) is a self‐reported screening tool composed of 27 items from the following areas: cognitive problems (13 items), hyperactive‐impulsive problems (10 items), and conduct problems (4 items) (Shin et al., 2001). Each item is scored on a three‐point scale; the total score ranges from 0 to 81, with high scores indicating a higher level of ADHD. Since the score for ADHD diagnosis varies depending on school grade, different scores were employed to identify ADHD: ≧ 41 for 7th and 8th graders, ≧ 44 for 9th graders, and ≧ 42 for 10th‐12th graders. The subjects whose scores satisfied the criteria were considered to exhibit ADHD proneness.

The BPAQ was used to evaluate levels of aggression among students (Buss & Perry, 1992). The rating scale had a four‐factor structure that included 29 items: physical aggression (9 items), verbal aggression (5 items), anger (7 items), and hostility (8 items). Each item is scored on a five‐point scale. The possible range for total score is 29 to 145, with high scores indicating higher levels of aggression. The CASS(S) and BPAQ were translated and revised in the Korean language (Cronbach's α = 0.901 and 0.913, respectively).

Demographic information included sex, age, body mass index (BMI), smoking status, alcohol consumption, parents’ attention, number of close friends, and academic performance. History of medical symptoms such as headache, muscle pain, scoliosis, gastrointestinal (GI) trouble, constipation, atopic dermatitis, sinusitis, asthma, unstable mood, fatigue or irritation, daytime sleepiness, and sleep induction time were also obtained.

### 2.3 Statistical analysis

A chi‐squared test was used to compare categorical variables such as demographic information and total aggression scores between participants with and without ADHD proneness. Since each of the subscales of aggression (physical aggression, verbal aggression, anger, hostility, and total aggression) was correlated with at least one other subscale, a multivariable analysis of variance (MANOVA), followed by a post hoc Tukey's‐b test, was conducted to examine differences in the subscales of aggression. To analyze the relationship between ADHD proneness and aggression propensity, Pearson's correlation coefficient was performed. A multivariable logistic regression analysis was conducted using the backward stepwise method, and area under the receiver operator characteristic (AUROC) curves were calculated to determine the cut‐off of total aggression scores for discriminating ADHD proneness. Odds ratio (OR) and adjusted OR (AOR) were estimated by univariate and multivariable analyses, respectively.

*P*‐values less than 0.05 were considered statistically significant. All statistical analyses were conducted with the Statistical Package for the Social Sciences version 20.0 for Windows (SPSS Inc., Chicago, Illinois, USA).

## 3 RESULTS

A total of 2,432 students participated in the survey, and 1,872 of them completed the questionnaire, for a response rate of 77.0%. Their mean age was 15.0 ± 1.8 years, and 891 (47.6%) were boys. In terms of school grades, 48.0% of the students were 7th‐9th graders (*n* = 898) and 52.0% were 10th‐12th graders (*n* = 974).

**FIGURE 1:**
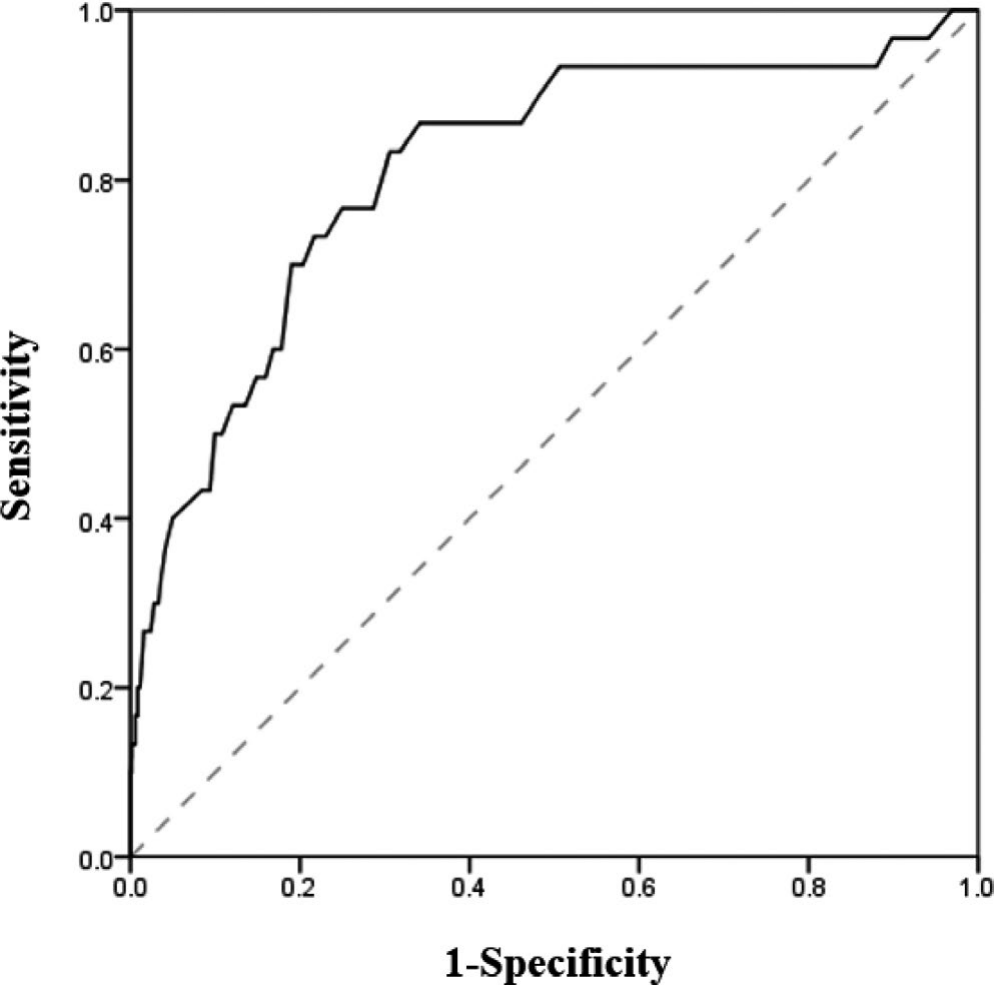
Area under the total aggression score curve (AUC) for ADHD proneness. The AUC value was 0.764 (95% CI 0.684–0.843, *p*‐value < 0.001), and the cut‐off value of total aggression was 68.5 points (sensitivity: 0.833, specificity: 0.694)

The AUROC curve analysis, which was performed to determine the cut‐off value for the total aggression score, showed that a score of 68.5 points had higher sensitivity (83.3%) and specificity (69.4%) to discriminate ADHD proneness (Figure 1). The AUROC value was 0.764 (95% CI 0.684–0.843).

A total of 33 (1.8%) adolescents were classified as the ADHD group based on CASS(S), and 57.6% of them were boys (Table 1). More than half of the adolescents in the ADHD group were 15 years or younger. Participants in the ADHD group tended to have a BMI in the top 10% of the study population, tobacco use, alcohol consumption, average or low parents’ attention, no or few friends, headache, muscle pain, scoliosis, GI trouble, constipation, and low academic performance (the lowest 25% in grade point average) compared with those in the non‐ADHD group. ADHD proneness was associated with mood in daily life, such as unstable mood, fatigue or irritation, daytime sleepiness, and total aggression score of 68.5 points or higher.

**TABLE 1:** Demographics of participants

		Control group (%)	ADHD group (%)	*P*
Sex				0.247
	Boys	872(47.4)	19(57.6)	
	Girls	967(52.6)	14(42.4)	
Age (years)				0.589
	≤15	1,083(59.2)	18(54.5)	
	>15	746(40.8)	15(45.5)	
High BMI				0.025
	Yes (BMI: top 10%)	158(9.8)	7(21.9)	
	No	1,451(90.2)	25(78.1)	
Smoking				0.001
	Yes	88(4.8)	6(18.2)	
	No	1735(95.2)	27(81.8)	
Alcohol				<0.001
	Yes	341(18.7)	15(45.5)	
	No	1,486(81.3)	18(54.5)	
Parents’ attention				0.001
	High	1,112(60.8)	11(33.3)	
	Average or Low	717(39.2)	22(66.7)	
Number of close friends				0.001
	Average or Many	1708(93.2)	26(78.8)	
	No or few	124(6.8)	7(21.2)	
Academic performance				0.002
	Average or High	1,453(79.8)	19(57.6)	
	Low (GPA: low 25%)	368(20.2)	14(42.4)	
Headache				0.010
	Yes	301(16.4)	11(33.3)	
	No	1538(83.6)	22(66.7)	
Muscle pain				<0.001
	Yes	345(18.8)	15(45.5)	
	No	1,494(81.2)	18(54.5)	
Scoliosis				0.013
	Yes	128(7.0)	6(18.2)	
	No	1711(93.0)	27(81.8)	
GI trouble				<0.001
	Yes	262(14.2)	14(42.4)	
	No	1577(85.8)	19(57.6)	
Constipation				0.041
	Yes	151(8.2)	6(18.2)	
	No	1688(91.8)	27(81.8)	
Atopic dermatitis				0.100
	Yes	260(14.1)	8(24.2)	
	No	1579(85.9)	25(75.8)	
Sinusitis				0.242
	Yes	169(9.2)	5(15.2)	
	No	1,670(90.8)	28(84.8)	
Asthma				0.177
	Yes	44(2.4)	2(6.1)	
	No	1795(97.6)	31(93.9)	
Unstable mood				0.001
	Yes	50(2.7)	4(12.1)	
	No	1789(97.3)	29(87.9)	
Fatigue or Irritation				<0.001
	Yes	334(18.2)	19(57.6)	
	No	1,500(81.8)	14(42.4)	
Daytime Sleepiness				<0.001
	Yes	504(27.4)	22(66.7)	
	No	1,333(72.6)	11(33.3)	
Sleep induction time (min)				0.061
	≤30	275(15.3)	9(27.3)	
	>30	1518(84.7)	24(72.7)	
Total aggression				<0.001
	≥68.5	538(30.6)	25(83.3)	
	<68.5	1,220(69.4)	5(16.7)	

Note

ADHD diagnosis criteria

‐ 1st or 2nd‐year student of middle school: CASS(S)≥41

‐ 3rd‐year student of middle school: CASS(S)≥44

‐ high school student: CASS(S)≥42

BMI, body mass index; CASS(S), Conners‐Wells’ Adolescent Self‐Report Scale (Short form); GI, gastrointestinal; GPA, grade point average.

As described in Table 2, high BMI, smoking and alcohol consumption, number of close friends, and academic performance had significant associations with all subdomains of ADHD. Medical conditions including headache, muscle pain, scoliosis, GI trouble, constipation, and sinusitis also showed a significant relationship with ADHD in all ADHD subdomains. Mood changes, such as unstable mood; fatigue or irritation; sleep disturbance, including daytime sleepiness and sleep induction time; and total aggression score of 68.5 points or higher were significantly associated with all ADHD subdomains.

**TABLE 2:** Univariate analysis of factors associated with ADHD proneness

		Conduct	Hyperactivity	Cognition	Total	*P*
Sex						<0.001
	Boys	2.91 ± 2.19***	4.71 ± 3.76***	9.12 ± 6.05	16.75 ± 10.57**	
	Girls	2.41 ± 2.07	3.79 ± 3.15	9.23 ± 5.89	15.42 ± 9.66	
Age (years)						
	≤15	2.74 ± 2.13*	4.24 ± 3.45	9.20 ± 5.87	16.19 ± 10.02	0.083
	>15	2.52 ± 2.16	4.22 ± 3.55	9.17 ± 6.12	15.91 ± 10.30	
High BMI						0.001
	Yes (BMI: top 10%)	3.25 ± 2.59***	4.83 ± 4.33*	10.42 ± 6.98**	18.50 ± 12.27**	
	No	2.59 ± 2.10	4.22 ± 3.47	9.03 ± 5.91	15.83 ± 10.04	
Smoking						<0.001
	Yes	3.62 ± 2.53***	6.63 ± 4.64***	11.10 ± 7.23**	21.34 ± 12.94***	
	No	2.60 ± 2.11	4.10 ± 3.37	9.08 ± 5.89	15.77 ± 9.90	
Alcohol						<0.001
	Yes	3.14 ± 2.35***	5.47 ± 4.08***	10.33 ± 6.58***	18.94 ± 11.31***	
	No	2.54 ± 2.08	3.93 ± 3.26	8.91 ± 5.79	15.38 ± 9.70	
						
Parents’ attention						<0.001
	High	2.73 ± 2.07	4.03 ± 3.20**	8.64 ± 5.73***	15.39 ± 9.55***	
	Average or Low	2.53 ± 2.25	4.55 ± 3.87	10.04 ± 6.23	17.12 ± 10.89	
Number of close friends						<0.001
	Average or Many	2.54 ± 2.05***	4.13 ± 3.44***	8.85 ± 5.76***	15.51 ± 9.81***	
	No or few	4.09 ± 2.71	5.58 ± 3.78	13.54 ± 6.94	23.21 ± 11.47	
Academic performance						<0.001
	Average or High	2.58 ± 2.13*	3.95 ± 3.34***	8.40 ± 5.67***	14.93 ± 9.71***	
	Low (GPA: low 25%)	2.90 ± 2.18	5.29 ± 3.84	12.15 ± 6.14	20.33 ± 10.54	
Headache						<0.001
	Yes	3.23 ± 2.46***	4.76 ± 3.77**	11.03 ± 6.84***	19.02 ± 11.42***	
	No	2.53 ± 2.05	4.12 ± 3.42	8.81 ± 5.71	15.46 ± 9.74	
Muscle pain						<0.001
	Yes	3.10 ± 2.34***	4.90 ± 3.97***	10.74 ± 6.69***	18.74 ± 11.51***	
	No	2.54 ± 2.08	4.07 ± 3.34	8.81 ± 5.72	15.41 ± 9.66	
Scoliosis						0.002
	Yes	3.19 ± 2.81**	5.14 ± 4.44**	10.87 ± 7.43**	19.21 ± 13.19***	
	No	2.60 ± 2.08	4.16 ± 3.39	9.05 ± 5.82	15.81 ± 9.81	
GI trouble						<0.001
	Yes	3.22 ± 2.57***	5.07 ± 4.34***	11.55 ± 6.97***	19.83 ± 12.50***	
	No	2.55 ± 2.04	4.08 ± 3.29	8.77 ± 5.68	15.40 ± 9.50	
Constipation						<0.001
	Yes	3.18 ± 2.54**	4.76 ± 4.14*	11.55 ± 6.76***	19.49 ± 11.89***	
	No	2.60 ± 2.10	4.18 ± 3.42	8.96 ± 5.84	15.74 ± 9.89	
Atopic dermatitis						0.063
	Yes	2.86 ± 2.27	4.48 ± 3.81	10.01 ± 5.90*	17.35 ± 10.61*	
	No	2.61 ± 2.12	4.19 ± 3.43	9.04 ± 5.97	15.84 ± 10.02	
Sinusitis						0.007
	Yes	3.09 ± 2.09**	4.87 ± 3.74*	10.53 ± 5.73**	18.49 ± 10.20**	
	No	2.60 ± 2.14	4.16 ± 3.45	9.04 ± 5.97	15.80 ± 10.08	
Asthma						0.054
	Yes	3.46 ± 2.59**	4.54 ± 3.89	9.63 ± 5.65	17.63 ± 10.69	
	No	2.62 ± 2.13	4.22 ± 3.47	9.17 ± 5.98	16.01 ± 10.11	
Unstable mood						<0.001
	Yes	4.87 ± 3.17***	5.63 ± 4.78**	14.22 ± 7.63***	24.72 ± 13.65***	
	No	2.58 ± 2.07	4.19 ± 3.43	9.03 ± 5.85	15.80 ± 9.89	
Fatigue or Irritation						<0.001
	Yes	3.61 ± 2.65***	5.77 ± 4.35***	12.71 ± 6.99***	22.09 ± 12.25***	
	No	2.42 ± 1.94	3.87 ± 3.15	8.37 ± 5.39	14.67 ± 9.10	
Daytime Sleepiness						<0.001
	Yes	3.10 ± 2.36***	5.42 ± 4.04***	11.85 ± 6.58***	20.37 ± 11.29***	
	No	2.47 ± 2.02	3.76 ± 3.12	8.14 ± 5.36	14.38 ± 9.09	
Sleep induction time (min)						<0.001
	≤30	3.42 ± 2.34***	5.23 ± 3.87***	11.09 ± 6.36***	19.74 ± 10.99***	
	>30	2.50 ± 2.08	4.03 ± 3.39	8.80 ± 5.82	15.33 ± 9.83	
Total aggression						<0.001
	≥68.5	3.52 ± 2.33***	6.22 ± 3.94***	12.39 ± 6.26***	22.13 ± 10.87***	
	<68.5	2.23 ± 1.89	3.29 ± 2.72	7.71 ± 5.16	13.22 ± 8.18	

Abbreviations

BMI, body mass index; CASS(S); GI, gastrointestinal; GPA, grade point average.

* *p* < .05

** *p* < .01

*** *p* < .001

The correlation analysis showed that students with ADHD proneness tended to have aggression propensity (Table 3). Among the aggression subdomains, anger and hostility had strong associations with ADHD proneness; Pearson's coefficients between ADHD and physical aggression, verbal aggression, anger, hostility, and total aggression were 0.348, 0.258, 0.473, 0.496, and 0.512, respectively.

**TABLE 3:** Correlation Analysis of ADHD proneness and aggressive propensity

	Cognition	Hyperactivity	Conduct	Total ADHD
Physical aggression	0.270***	0.387***	0.267***	0.348***
Verbal aggression	0.209***	0.271***	0.204***	0.258***
Anger	0.286***	0.442***	0.442***	0.473***
Hostility	0.367***	0.379***	0.488***	0.496***
Total aggression	0.362***	0.473***	0.458***	0.512***

Note

Total ADHD was assessed using Revised short form of the Conners‐Wells Adolescent Self‐Report Scale.

Total aggression was evaluated using Buss‐Perry Aggression Questionnaire.

*** *p* < .001

**FIGURE 2:**
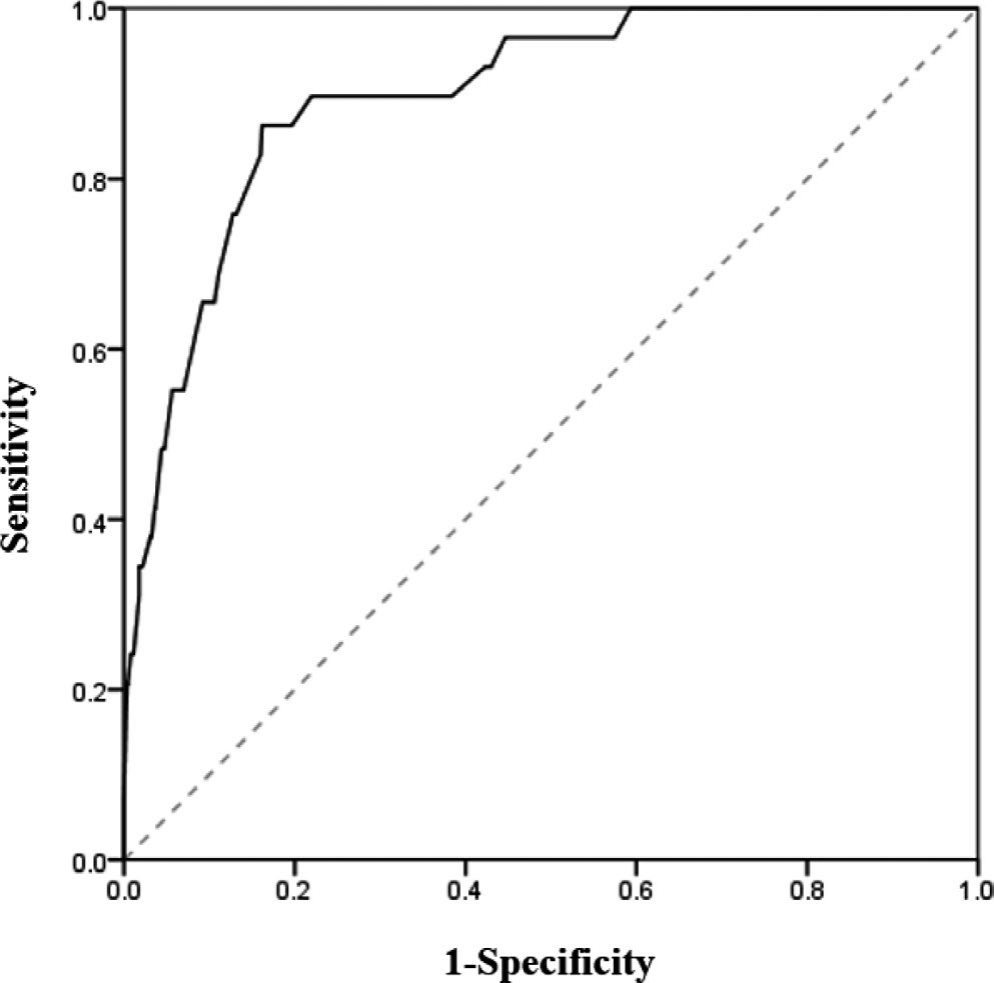
Area under receiver operating characteristic curve for ADHD proneness that included high BMI, parents’ attention, number of close friends, academic performance, alcohol consumption, smoking status, headache, muscle pain, scoliosis, GI trouble, constipation, unstable mood, fatigue or irritation, daytime sleepiness, and total aggression score (≥ 68.5) for analysis

To assess the risk of ADHD in relation to total aggression scores, a multivariable analysis was conducted (Table 4). Students in the top 10% of BMI and with alcohol consumption had 3.0‐ and 2.9‐fold higher risks of ADHD proneness, respectively, and the presence of GI trouble and daytime sleepiness increased the risk of ADHD proneness by 3.7‐ and 4.7‐fold, respectively. Participants with a total aggression score of 68.5 or higher had a 9.8‐fold higher risk of ADHD than those with a total aggression score less than 68.5. The Hosmer–Lemeshow test revealed a good fit (x^2^ = 5.679, *p = 0.460*), and the AUROC was 0.897 (95% CI 0.845–0.949; Figure 2).

**TABLE 4:** Multivariable analysis for predictive factors of ADHD

Factors	Unadjusted OR	Adjusted OR
	(95% CI)	(95% CI)
High BMI (BMI: top 10%)	2.571(1.095–6.041)*	3.043(1.166–7.939)*
Parents’ attention (High)	0.322(0.155–0.669)**	
Number of close friends (Average or Many)	0.270(0.115–0.634)**	
Academic performance (Average or High)	0.344(0.171–0.692)**	
Alcohol	3.631(1.812–7.278)***	2.855(1.300–6.269)**
Smoking	4.381(1.763–10.886)**	
Headache	2.555(1.226–5.324)*	
Muscle pain	3.609(1.801–7.232)***	
Scoliosis	2.970(1.205–7.326)*	
GI trouble	4.435(2.197–8.955)***	3.705(1.649–8.324)**
Constipation	2.484(1.010–6.111)*	
Unstable mood	4.935(1.672–14.569)**	
Fatigue or Irritation	6.095(3.025–12.279)***	
Daytime Sleepiness	5.290(2.547–10.987)***	4.664(1.988–10.943)***
Total aggression (≥68.5)	11.338(4.317–29.776)***	9.798(3.330–28.832)***

Abbreviations

BMI, body mass index; CI, confidence interval; GI, gastrointestinal; OR, odds ratio.

* *p* < .05

** *p* < .01

*** *p* < .001

## 4 DISCUSSION

This study showed a significant association between adolescent ADHD proneness and aggression propensity. Among the subdomains of aggression, anger and hostility had stronger associations with ADHD proneness than did physical and verbal aggression.

A recent study has reported that psychiatric disorders, especially ADHD, occur simultaneously with aggression in pediatric patients (Bartels et al., 2018). Our study also showed that ADHD proneness increased as the total aggression score increased, indicating that ADHD tendency and aggression propensity had a significant association; the prediction model showed a good ability to predict this association, with an AUROC value of 0.897.

Anger and hostility indicate the affective and cognitive facets of aggression, while physical and verbal aggression constitute the instrumental facets of aggression (Reyna et al., 2011; Chung et al., 2019). Adolescents with ADHD, unlike children with ADHD, show less hyperactivity and more internal problems, such as frustration and low self‐esteem (Cantwell, 1996; Dupaul, 1991; Jeong et al., 2001). These psychological difficulties are known to be caused by an inadequate understanding of ADHD‐related problem behaviors by parents or teachers, and by a negative evaluation from peer groups (Erk, 1997; Park & Chun, 2018). The repetition of frustrating situations and lack of self‐esteem lead to anger and hostility, and the rapid physiological changes of the adolescent period can amplify this internal aggression (Houston & Vavak, 1991; Spielberger et al., 1983). Our findings that hostility and anger were more strongly correlated with ADHD proneness than were the other subdomains of aggression are reasonable results based on the characteristics of adolescent ADHD.

The relationship between aggression and ADHD in adolescents is different from that in childhood. Hyperactivity is the most prominent among ADHD symptoms in childhood but decreases in adolescence (Fischer et al., 2005; Hart et al., 1995). A follow‐up result of adolescent ADHD patients who were diagnosed with ADHD and CD in childhood showed that anger was a more persistent symptom than physical aggression (Harty et al., 2009). Therefore, it was speculated that emotional dysregulation in adolescent ADHD patients is an important factor for aggression.

Among baseline characteristics, obesity, alcohol consumption, GI trouble, and sleep problem were significantly associated with ADHD proneness in this study. The potential association between obesity and ADHD has already been reported in a meta‐analysis (Cortese et al., 2008). However, the causal relationship has been unclear because of inconsistent study results. Patients with ADHD had a high possibility of experiencing comorbid illnesses (Ohlmeier et al., 2008; Jameson et al., 2016). Among comorbidity issues, abuse problems, including alcoholism, were found in 35%–70% of patients with ADHD (Ohlmeier et al., 2008). In addition, serious stomach or bowel problems had a strong association with ADHD (Jameson et al., 2016). In terms of sleep problems, meta‐analyses have reported that daytime sleepiness is more prevalent in children with ADHD than in typical children (Cortese et al., 2006; Sadeh et al., 2006). Studies of children with ADHD have demonstrated difficulty with sleep; this trouble would naturally be related to daytime sleepiness (Crabtree et al., 2003; Spruyt & Gozal, 2011).

A limitation of this study relates to its cross‐sectional design. In addition, the prevalence rate of adolescent ADHD in our study was relatively lower than that of previous studies from 7.2% to 9.4% (Willcutt, 2012; Danielson et al., 2018). This may possibly be due to the nature of a self‐reported instrument, which is the type of tool used in this study, as self‐reported assessments may accompany social desirability bias. Nevertheless, in the absence of research on ADHD in adolescents, this study was conducted on a large scale of 1,900 adolescents. Overall, our study demonstrated a significant association between ADHD proneness and aggression propensity. Among subdomains of aggression, anger and hostility showed a strong correlation with ADHD tendency. Physical and verbal aggression are overt aggressive behaviors, but hostility and anger are inconspicuous behaviors that are difficult for teachers, parents, and others to detect. Accordingly, public awareness of ADHD‐associated aggression factors should be increased, and comprehensive prevention efforts should be implemented by government agencies and schools.

## CONFLICT OF INTEREST

The authors declare no conflict of interest.

## AUTHOR CONTRIBUTION

HJY, JMH and HSG have made substantial contributions to conception and design. Acquisition of data were performed by HJY, JMH and KK. Data analysis and interpretation were performed by GS, JY, JEC, and KEL. HJY and JMH have been involved in drafting the manuscript. The manuscript was revised by HSG. All authors reviewed and approved the final manuscript.

### Peer Review

The peer review history for this article is available at https://publons.com/publon/10.1002/brb3.2030.

## Data Availability

The data that support the findings of this study are available from the corresponding author upon reasonable request.
